# Baicalein ameliorates TNBS-induced colitis by suppressing TLR4/MyD88 signaling cascade and NLRP3 inflammasome activation in mice

**DOI:** 10.1038/s41598-017-12562-6

**Published:** 2017-11-27

**Authors:** Xiaoping Luo, Zhilun Yu, Chao Deng, Jingjing Zhang, Gaiyan Ren, Aning Sun, Sridhar Mani, Zhengtao Wang, Wei Dou

**Affiliations:** 1 Shanghai Key Laboratory of Formulated Chinese Medicines, Institute of Chinese Materia Medica, Shanghai University of Traditional Chinese Medicine, Shanghai, 201203 China; 2 Departments of Medicine and Genetics, Albert Einstein College of Medicine, Bronx, New York 10461 USA

## Abstract

Baicalein (5,6,7-trihydroxyflavone), a predominant bioactive component isolated from the root of *Scutellaria baicalensis* Georgi, has established potent anti-inflammatory activity via multi-targeted mechanisms. However, little is known about the effect of baicalein on 2,4,6-trinitrobenzene sulfonic acid (TNBS)-induced colitis, which shares pathology related to human Crohn’s disease (CD). The present study demonstrated that baicalein alleviated the severity of TNBS-induced colitis in mice by decreasing the activity of myeloperoxidase (MPO) and the expression of pro-inflammatory mediators. The decline in the activation of nuclear factor-kappa B (NF-κB) and p38 mitogen-activated protein kinase (MAPK) correlated with a decrease in the expression of mucosal toll-like receptor 4 (TLR4) and its adaptor myeloid differentiation factor 88 (MyD88). *In vitro*, baicalein down-regulated the TLR4/MyD88 signaling cascades (NF-κB and MAPKs) in lipopolysaccharide (LPS)-stimulated macrophages. At the upstream level, baicalein bound to the hydrophobic region of the myeloid differentiation protein-2 (MD-2) pocket and inhibited the formation of the LPS-induced MD-2/TLR4 complex. Furthermore, baicalein reduced NOD-like receptor 3 (NLRP3) inflammasome activation and downstream interleukin-1β expression in a dose-dependent manner. Our study provided evidence for the first time that baicalein attenuated TNBS-induced colitis, at least in part, via inhibition of TLR4/MyD88 signaling cascade as well as inactivation of NLRP3 inflammasome.

## Introduction

Inflammatory bowel disease (IBD), a group of chronic and relapsing intestinal inflammatory disorders, typified by Crohn’s disease (CD) and ulcerative colitis (UC), remains incurable at present. Despite a plethora of drugs available to treat these conditions, due to numerous side effects and lack of sustained efficacy, two-thirds of patients with CD and one-third of patients with UC eventually need surgery at certain point during the course of disease^1,2^. In terms of quality of life, non-toxic and non-mutagenic therapies, especially those derived from natural products, are gaining considerable interest in the scientific community^3,4^.

Baicalein (5,6,7-trihydroxyflavone) is a principle bioactive flavonoid isolated from the roots of *Scutellaria baicalensis* Georgi, a traditional herbal medicine used to treat various types of inflammatory diseases^5^. Our group have previously reported the potent anti-inflammatory activity of baicalein on dextran sodium sulfate (DSS)-induced colitis in mice, a well-established experimental model with features resembling human UC, via targeting to caudal-type homeobox 2 (CDX2/pregnane X receptor (PXR) pathway^6^. In this regard, other mechanisms implicated in the actions of baicalein on DSS-induced colitis include reduced peroxisome proliferator-activated receptor-γ (PPARγ) signaling^7^. In addition, baicalin is the 7-glucuronic acid conjugate of baicalein^6^. The effects of baicalin on rodent colitis are better addressed to act via abrogation of oxidant stress^8^, T-helper 17 (Th17)/regulator T cell (Treg) balance^9^, toll-like recptor 4 (TLR4)/nuclear factor kappa B (NF-κB) signaling^10^, and macrophage inhibitor factor (MIF) signaling^11^. Other studies have examined cocktails of extracts (e.g. PF2405) that do not specifically address mechanisms of action of individual flavonoids in *S. baicalensis* ^12,13^. Nevertheless, the direct effects of baicalein on 2,4,6-trinitrobenzene sulfonic acid (TNBS)-induced colitis, a model simulating human CD, and the underlying mechanisms remain unknown.

In this study, we demonstrated that baicalein exerted significant anti-inflammatory activity in TNBS-induced colitis in mice possibly via abrogating TLR4/myeloid differentiation factor 88 (MyD88) signaling pathway and its downstream signaling molecules, NF-κB and mitogen-activated protein kinases (MAPKs). In addition, the nucleotide-binding oligomerization domain (NOD)-like receptor (NLR) pyrin domain containing 3 (NLRP3) inflammasome, which was required for the secretion of interleukin 1β (IL-1β), was also abrogated by baicalein.

## Materials and Methods

### Cell lines and reagents

THP-1 human macrophage cell line and RAW264.7 mouse macrophage cell line were obtained from the American Type Culture Collection (Manassas, VA, USA). All cells were cultured in Dulbecco’s modified Eagle’s medium supplemented with 10% fetal bovine serum and a mixture of antibiotics (100 units/ml penicillin and 100 μg/ml streptomycin) under 5% CO_2_ at 37 °C. Baicalein (Lot No. 05-2001, HPLC purity ≥98%) was kindly provided by the Shanghai R&D Center for the Standardization of Traditional Chinese Medicine (Shanghai, China). Dual-Luciferase reporter assay system and 1 × Passive Lysis Buffer were form Promega (Medison, WI). Antibodies for inducible nitric oxide synthase (iNOS, #13120), cyclooxygenase-2 (COX-2, #12282), extracellular signal-regulated kinase 1/2 (ERK1/2, #4348), p-ERK1/2 (#4377), c-Jun N-terminal kinase (JNK, #9255), p-JNK (#9252) ,p38 (#9212), p-p38 (#9215), myeloid differentiation protein-2 (MD-2), NF-κB p65, p-p65, IκBα, p-IκBα, IL-1β and β-actin (#4970) were obtained from Cell Signaling Technology (Danvers, MA, USA). Antibodies for MyD88 (AP8521C), IL-1 phospho-receptor-associated kinase 1 (p-IRAK1, AP50215) were obtained from Abgent (San Diego, CA, USA). Antibodies for TLR4 (ab13556), caspase-1 (ab1872), NLRP3 (ab214185) and apoptosis-associated speck-like protein containing a caspase recruitment domain (ASC, ab47092) were from Abcam (Cambridge, MA, USA). Alexa Fluor 488-conjugated secondary antibody (A21206), lipofectamine 2000 transfection reagent, Triton X-100, Trizol, DAPI and the SuperScript II Reverse Transcriptase kit were from Thermo Scientific Inc. (Waltham, MA, USA). SYBR Premix ExTaq Mix was from Takara Biotechnology (Shiga, Japan). The Myeloperoxidase (MPO) activity assay kit and the nitric oxide (NO) assay kit were from Nanjing Jiancheng Bioengineering Institute (Nanjing, China). Cell counting kit 8 (CCK-8) assay kit was from Dojindo Laboratories, Japan. Donkey serum, TNBS, lipopolysaccharide (LPS), DEPC water, formalin, paraformaldehyde, Tween-20, ethanol, dimethyl sulphoxide (DMSO), diaminobenzidine were from Sigma-Aldrich (St Louis, MO, USA). The enhanced chemiluminescence (ECL) detection kit was from Millipore (Billerica, MA).

**Mice**. Healthy 8-week-old female Balb/c mice (20 ± 2 g) were obtained from the Shanghai Laboratory Animal Center, and the subsequent studies were performed in accordance with the guidelines approved by the Animal Ethics Committee of Shanghai University of Traditional Chinese Medicine (SHUTCM). Standard mouse chow pellets and water were supplied *ad libitum*. All mice were housed under a specific pathogen-free facility at SHUTCM and kept under the same temperature (25 ± 2 °C) and lighting (12-h light-dark cycle) conditions.

### *In vivo* study

#### TNBS-induced colitis

TNBS-colitis was induced in mice as described previously^14,15^. The experiment lasted for 10 days. A baicalein stock solution was prepared in 0.5% methylcellulose and administered to mice at a dose of 20 mg/kg/d by oral gavage. Baicalein dosing (20 mg/kg/d per body weight) was based on previous reports by we and others^6,16^. 8-week-old female Balb/c mice were randomly distributed into the following four groups (n = 10 mice per group): Group 1 comprised the vehicle controls, which were administered 100 µl of 0.5% methylcellulose by oral gavage once per day; Group 2 comprised baicalein treated mice at a dose of 20 mg/kg of body weight via oral gavage once per day; Group 3, 100 µl of 0.5% methylcellulose by oral gavage once per day and 2 mg (in 100 μl of 50% ethanol) of TNBS (Sigma-Aldrich, St.Louis, MO) was administered intrarectally to fasted and anesthetized mice via a catheter inserted 3 cm proximally to the anus on day 3; Group 4, received baicalein by oral gavage 2 days prior to TNBS administration and continued to the end of the study (d 10).

#### Clinical and histological assessment of colitis

Mice were monitored daily for body weight, diarrhea and bloody stool incidence. mice were sacrificed under anesthesia 4 h after receiving the last gavage. The entire colon was removed and the total length was measured. The entire colon was fixed in 10% buffered formalin for 24 h at room temperature, embedded in paraffin and stained with hematoxylin-eosin (H&E) for histological evaluation. Histological damage was assessed as a combined score of inflammatory cell infiltration (score 0–3) and mucosal damage (score 0–3) using a previously described method^14,15^. Briefly, for inflammatory cell infiltration in the colon mucosa, rare inflammatory cells (mononuclear infiltrates) in the lamina propria were counted as 0; increased numbers of inflammatory cells, including neutrophils in the lamina propria as 1; confluence of inflammatory cells, extending into the submucosa as 2; and a score of 3 was given for transmural extension of the inflammatory cell infiltration. For epithelial damage, absence of mucosal damage was counted as 0; discrete focal lymphoepithelial lesions were counted as 1; mucosal erosion/ulceration was counted as 2; and a score of 3 was given for extensive mucosal damage and extension through deeper structures of the bowel wall. The two sub-scores were added and the combined histologic score ranged from 0 (no changes) to 6 (extensive cell infiltration and tissue damage).

#### RNA analysis

RNA was extracted using TRIzol reagent. Quantitative real-time polymerase chain reaction (qPCR) was performed using cDNA generated from 3 μg of total RNA with the SuperScript II Reverse Transcriptase kit. The following PCR primer sequences were used: 5′-GGGAATCTTGGAGCGAGTTG-3′/5′-GTGAGGGCTTGGCTGAGTGA-3′ for iNOS, 5′-CGCTGTGCTTTGAGAACTGT-3′/5′-AGGTCCTTGCCTACTTGCTG-3′ for intercellular adhesion molecule-1 (ICAM-1), 5′-GAAGTCTTTGGTCTGGTGCCT-3′/5′-GCTCCTGCTTGAGTATGTCG-3′ for COX-2, 5′-AAGTTGACCCGTAAATCTGA-3′/5′-TGAAAGGGAATACCATAACA-3′ for monocyte chemotactic protein-1 (MCP-1), 5′-GTTCTGCCATTGACCATCTC-3′/5′-TGATACTGTCACCCGGCTCT-3′ for IL-1α, 5′-GGCTGGACTGTTTCTAATGC-3′/5′-ATGGTTTCTTGTGACCCTGA-3′ for IL-1β, 5′-CGTGGAACTGGCAGAAGAGG-3′/5′-AGACAGAAGAGCGTGGTGGC-3′ for tumor necrosis factor-α (TNF-α), and 5′-CAGCCTTCCTTCTTGGGTAT-3′/5′-TGGCATAGAGGTCTTTACGG-3′ for β-actin. PCR reactions were carried out using SYBR Premix ExTaq Mix and quantitatively measured with an ABI Prism 7900HT Sequence Detection System (Life technologies, Carlsbad, CA). The following thermal cycler parameters were used: 1 cycle of 95 °C for 30 s and 40 cycles of denaturation (95 °C, 5 s) and combined annealing/extension (60 °C, 30 s). Gene expression changes were calculated by the comparative Ct method, and the values were normalized to the β-actin endogenous reference.

#### Myeloperoxidase (MPO) assay

Tissue MPO activity, which is linearly related to neutrophil infiltration in inflamed tissue, was determined as described previously^17^. The colonic tissues were weighed and homogenized. The supernatants were collected. The activity of MPO in the supernatants was measured using a detection kit according to the manufacturer’s instructions.

#### Immunohistochemistry

The paraffin-embedded colonic tissue slides were incubated with antibodies against mouse phospho-NF-kB p65-NLS as described previously^17^. After further washing, the slides were incubated with Envision/HRP at 37 °C for 30 min. Finally, immune complexes were visualized by incubating with diaminobenzidine for 10 min and counterstained with hematoxylin.

### *In vitro* study

#### Cell viability assay

Raw264.7 cells (2 × 10^5^/well) were seeded in 96-well plate. After overnight incubation. Cell viability was determined using a CCK-8 assay kit. 10ul of CCK-8 solution was added to each well and incubated for 30 min at 37 °C. Absorbance was measured at 450 nm using a spectrophotometer.

#### Immunoblotting

Total proteins extracted from colon tissues and cultured cells were lysed, homogenized and centrifuged. The protein content in the supernatants was measured using the BCA kit. 10–30 μg of protein was separated by 10% SDS-PAGE and transferred onto a nitrocellulose membrance. After blocking with 5% skim milk, the membranes were incubated with antibodies against iNOS, COX-2, TLR-4, MyD88, NF-κB p65, p-p65, IκBα, p-IκBα, ERK1/2, p-ERK1/2, JNK, p-JNK, p-38, p-p38, p-IRAK1, NLRP3, ASC, caspase-1, IL-1β and β-actin, respectively. The membranes were then incubated with the specific secondary antibodies conjugated to horseradish peroxidase (HRP). The blots were developed using an ECL detection kit. The protein bands were analyzed using a GS-700 imaging densitometer (Bio-Rad, CA) and quantified following normalization to the expression of β-actin.

#### NO assay

The production of NO in the supernatant was determined using a NO assay kit as described previously^17^.

#### NF-κB luciferase reporter assay

RAW264.7 cells were seeded in a 96-well plate at a density of 2 × 10^5^ cells/well one day before transfection. The cells were transfected with 0.8 µg of the NF-kB reporter vector pGL4.32[luc2P/NF-kB-RE/Hygro] (Promega, Madison, WI) using the lipofectamine 2000 reagent as described previously^18^. Cells were pretreated with baicalein (10, 25 and 50 μM) for 2 h and then stimulated with LPS (1 μg/ml) for 48 h. Cells were washed once with PBS and lysed in 100 µl of 1 × passive lysis buffer. Cell-free lysates were obtained by centrifugation at 10,000 *g* for 2 minutes at 4 °C. Luciferase activity from cell lysates was quantified using a luciferase assay system and a Glomax 20/20 luminometer (Promega, Madison, WI, USA). Results were expressed as fold induction of control cells.

#### Immunofluorescence staining

The immunostaing was performed as described previously^17^. Briefly, RAW264.7 cells were fixed in 4% (w/v) paraformaldehyde for 10 min at room temperature, and washed three times with PBS, then permeabilized with 0.3% (w/v) Triton X-100 for 20 min at room temperature, followed by three washes with PBS. After incubation in PBS containing 10% bovine serum albumin for 30 min at room temperature, the slides were incubated with antibody against p-p65-NLS (PA523170, Thermo Scientific Inc., Waltham, MA, USA) overnight at 4 °C and then incubated with Alexa Fluor 488-conjugated secondary antibody for 1 h in the dark. To stain the nuclei, 1 µg/ml of 4′,6-diamidino-2-phenylindole in PBS was added before capturing images with a fluorescence microscope (Olympus CKX41, Tokyo, Japan).

#### Molecular docking

Molecular docking of baicalein with myeloid differentiation protein-2 (MD-2) was carried out using MOE (Molecular Operating Environment Program, version 2016.08, Chemical Computing Group, Montreal, Canada). The available three dimensional structures of MD-2 was obtained from the Protein Data Bank (PDB code: 2E56, Resolution: 2.0 Å). The co-crystalized structure was prepared using QuickPrep for correcting structure errors (e.g. broken bonds, missing loops, empty residues, etc.), adding hydrogen, and calculating partial charge. The 2D structure of baicalein was downloaded from the PubChem database with SD file format and converted to 3D in MOE through energy minimization. The hydrophobic region of MD-2 was chosen as the binding pocket for docking. Classical Triangle Matching was selected for the placement method and the number of placement poses was set to 100. The output docking poses were evaluated by the London dG score. The Rigid Receptor Method which keeps ligand-binding groove rigid was employed in the refinement step. The number of the final docking poses was set to 50, followed by minimizing using Amber10: EHT force field in MOE. The GBVI/WSA dG score was used to evaluate the binding affinity. The binding mode and the ligand-protein interactions were analyzed using MOE after minimization.

#### Immunoprecipitation

The assay was performed using a protein A immunoprecipitation kit (Cat No. 10006D, Life technologies, Carlsbad, CA) according to the manufacturer’s instructions. 500 μg cellular protein was incubated with 2 μg anti-TLR4 antibody for 2 h at 4 °C and then immunoprecipitated with 20 μl protein A beads at 4 °C overnight. The immunoprecipitates were washed 3 times with IP buffer (supplied in kit) and subjected to immunoblotting with anti-MD2 antibody and anti-TLR4 antibody, respectively.

#### Statistics

All data were expressed as the mean ± SD. The differences between groups were analyzed by one-way analysis of variance (ANOVA) followed by the least significant difference (LSD) for post-hoc test. Statistical analysis was performed by the SPSS 16.0 software package. A value of p < 0.05 was considered statistically significant.

## Results

### *In vivo* study

#### Baicalein treatment attenuated TNBS-induced colitis

**Figure 1:**
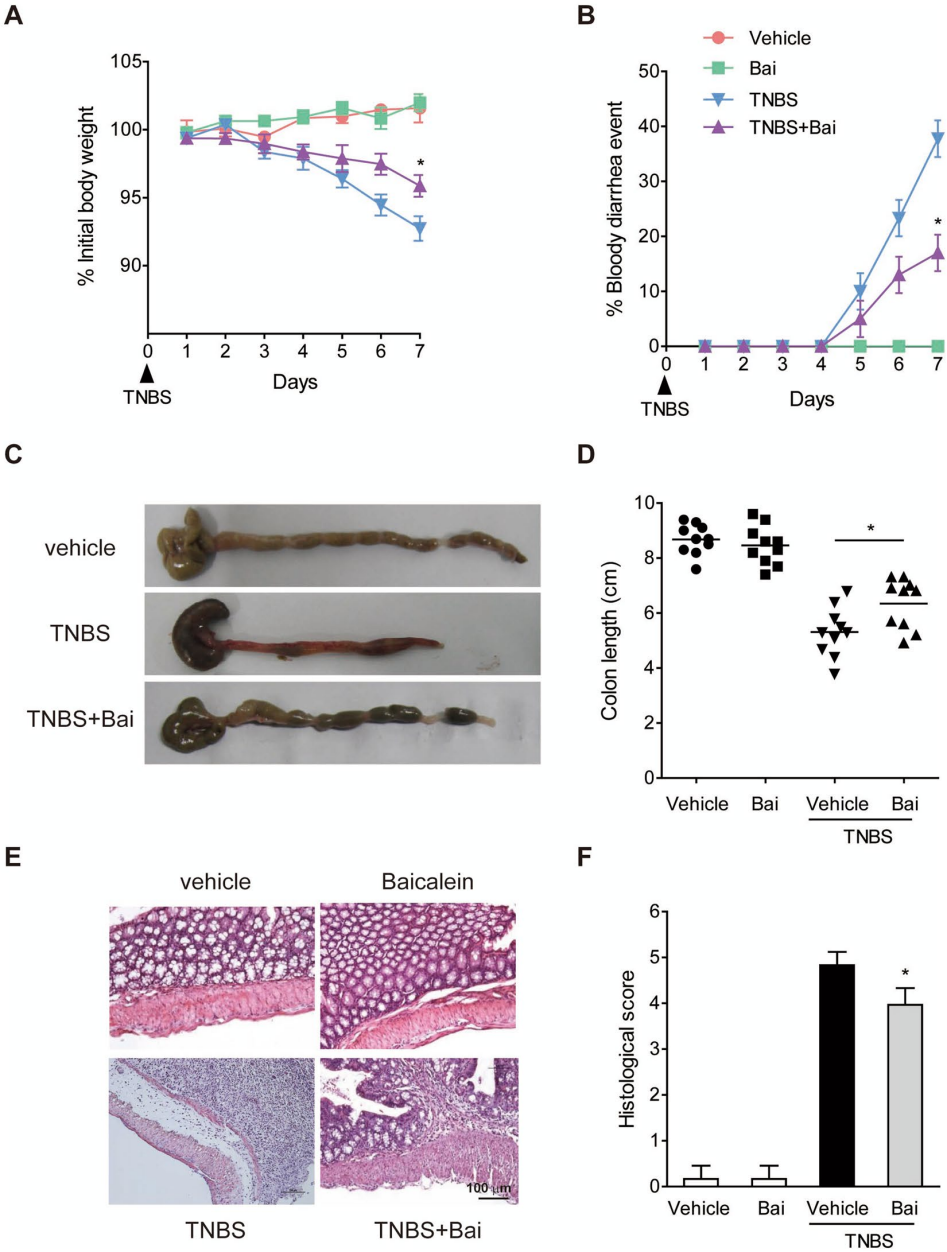
Baicailein ameliorated the progression TNBS-induced colitis in mice. (**A**) Body weight changes following TNBS induction of colitis. Data were plotted as the percentage of the basal body weight. (**B**) The occurrence of bloody diarrhea. Data were plotted as the percentage of total mice that had bloody diarrhea at different time points of TNBS treatment. (**C**) A representative view of the colon morphology. (**D**) Colon length was measured at the end of the study. (**E**) Representative H&E-stained colon sections. Scale bar corresponds to 100 μm and applies throughout. (**F**) Histological score. Values were expressed as the mean ± SD (n = 8). *p < 0.05 vs. TNBS-treated group.

Rectal administration of TNBS induced severe colitis in mice that was characterized by weight loss and diarrhea. Administrtion of baicalein significantly reduced body weight loss and bloody diarrhea symptoms (Fig. 1A,B). Colon shortening is an indirect marker of inflammation^19^. As expected, the TNBS-induced colon shortening was improved by baicalein treatment (Fig. 1C,D). Histologically, the colonic tissues of TNBS-treated mice showed marked crypt destruction, mucosal ulceration, and inflammatory cell infiltration. These parameters were attenuated in mice receiving baicalein treatment (Fig. 1E,F). In addition, there was no weight loss, diarrhea, colon shortening or mucosal disruption observed in control mice receiving vehicle or baicalein alone during the study.

#### Baicalein decreased the activity of MPO

**Figure 2:**
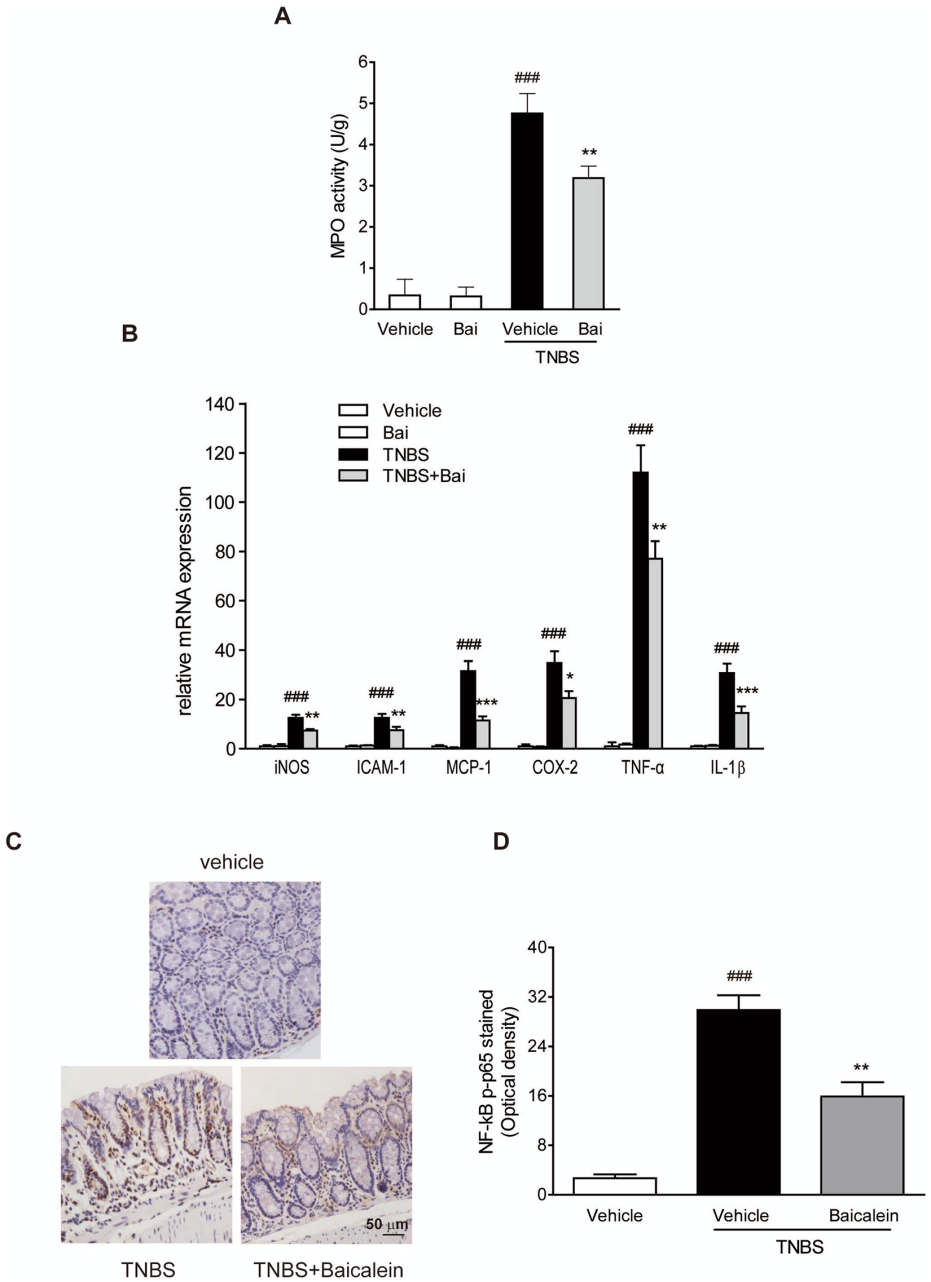
Baicalein inhibited NF-κB pathway in TNBS-induced mice. (**A**) Colon segments from mice were excised and homogenized. The supernatants were assayed for the determination of the activity of MPO. (**B**) mRNA expression of iNOS, ICAM-1, MCP-1, COX-2, TNF-α, and IL-1β in colonic tissue was determined by qRT-PCR. Expression was normalized to β-actin. (**C**) Representative images of p-p65 immunostaining in colonic tissue. Scale bar corresponds to 50 μm and applies throughout. (**D**) The mean intensity of p-p65 staining was determined by image analysis and was represented as optical density. Data were expressed as mean ± SD (n = 6). ^###^p < 0.001 vs. vehicle-treated group; *P < 0.05, **p < 0.01, ***P < 0.001 vs. TNBS-treated group.

MPO is an enzyme found in neutrophils and its activity is linearly related to neutrophil infiltration in inflamed tissue^18^. Mice treated with TNBS showed a significant increase in intestinal MPO activity compared to the control group, and baicalein treatment caused a significant inhibition of MPO activity (Fig. 2A).

#### Baicalein inhibited inflammatory mediator genes

To elucidate the underlying mechanisms of baicalein on TNBS-induced colitis, qRT-PCR analysis of several pro-inflammatory mediator genes in the colon was performed. mRNA expression of iNOS, ICAM-1, MCP-1, COX-2, TNF-α and IL-1β was remarkably induced in the colonic tissues of TNBS-colitis mice (Fig. 2B). Administration of baicalein greatly reduced the levels of the pro-inflammatory mediator genes in the inflamed colon.

#### Baicalein inhibited the activation of NF-κB and MAPK

**Figure 3:**
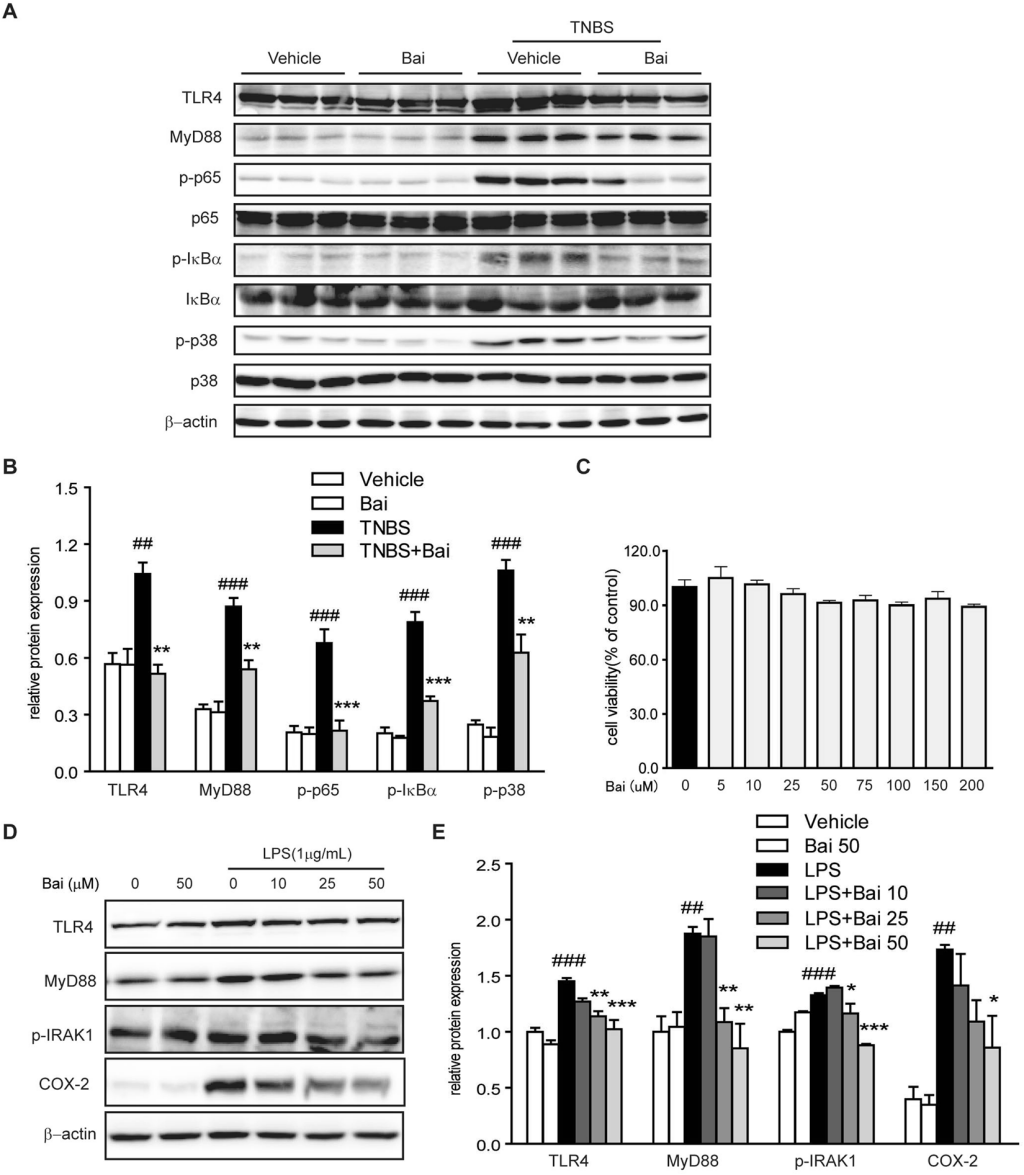
Baicalein inhibited TLR4/MyD88 signaling molecules *in vivo* and *in vitro*. (**A**) Mice were sacrificed at the end of the study, and total protein from the colon tissues was subjected to immunoblotting with antibodies against TLR4, MyD88, NF-κB p65, p-p65, IκBα, p-IκBα, p-38, p-p38 (1:1000 dilution) and β-actin (1:2000 dilution). One representative blot was shown. (**B**) Quantification of the protein expression was performed by densitometric analysis of the blots. (**C**) RAW264.7 cells were exposed to baicalein (0, 5, 10, 25, 50, 75, 100, 150 and 200 μM) for 48 h. Cell viability was determined using a CCK-8 assay kit. (**D**) RAW264.7 cells were treated with baicalein (0, 10, 25, 50 μM) for 2 h followed by an additional treatment with or without LPS (1 μg/ml) for 24 h. Total protein was extracted and subjected to immunoblotting with antibodies against TLR4, MyD88, p-IRAK-1, COX-2 (1:1000 dilution) and β-actin (1:2000 dilution). One representative blot was shown. (**E**) Quantification of the protein expression was performed by densitometric analysis of the blots. Expression was normalized to β-actin. Data were expressed as mean ± SD of three independent experiments (n = 3). ^##^p < 0.01, ^###^p < 0.001 vs. vehicle-treated group; *P < 0.05, **p < 0.01, ***P < 0.001 vs. TNBS/LPS-treated group.

The activation of NF-κB and MAPK pathways has been implicated in the pathogenesis of IBD^20^. We then evaluated the effects of baicalein on the activation of NF-κB and MAPK signaling molecules in TNBS-colitis mice. We performed immunostaining on paraffin-embedded colonic tissue using anti-p-p65 antibody, and the results showed that TNBS treatment led to a pronounced phosphorylation of NF-κB p65 and IκBα in the colonic tissue (Fig. 2C,D). However, baicalein administration significantly reduced the phosphorylation of p65 and IκBα in the colon of TNBS-colitis mice, which was in accord with the immunoblotting results (Fig. 3A and B). In addition, baicalein inhibited TNBS-induced phosphorylation (activation) of p-38 MAPK (Fig. 3A,B), but the phosphorylation levels of ERK1/2 and JNK did not changed by baicalein treatment (data not shown).

#### Baicalein inhibited the expression of TLR4 signaling molecules

Since TLR4 governs signals to NF-κB and MAPK via interacting with adaptor protein MyD88, we determined the effects of baicalein on the activation of TLR4 and MyD88 by immunoblotting. The protein expression of TLR4 and MyD88 in mice after TNBS treatment was up-regulated compared with the normal control mice (Fig. 3A,B). However, the relative increase in the expression of TLR4 and MyD88 after TNBS treatment was significantly down-regulated in mice subjected to baicalein treatment.

### *In vitro* study

#### Evaluation of the cytotoxic effects of baicalein on RAW264.7 cells

The cytotoxicity of baicalein was evaluated using the CCK-8 assay. RAW264.7 cells were incubated with baicalein at a wide range of concentrations (0, 5, 10, 25, 50, 75, 100, 150 and 200 μM). The data showed no significant changes in cell viability, indicating that baicalein was not cytotoxic at dosage up to 200 μM (Fig. 3C).

#### Baicalein decreased the activation of TLR4/MyD88 signaling molecules

To provide further insight into the mechanisms of baicalein, the activation of TLR4 signaling pathway in LPS-stimulated RAW264.7 macrophages was evaluated by immunoblotting. The results showed that the expression levels of TLR4 and its regulator molecule MyD88 were increased by LPS treatment, which were down-regulated by baicalein in a dose-dependent manner (Fig. 3D,E). Since activation of IRAK-1 plays a central role in TLR4 signaling cascade and increased level of phosphorylated IRAK-1 leads to the activation of NF-κB and MAPK pathways^21^, we then tested the effect of baicalein on the activation of IRAK-1. Baicalein inhibited the phosphorylation of IRAK-1 induced by LPS in a dose-dependent manner. Furthermore, the COX-2 protein level was also dose-dependently inhibited by baicalein treatment (Fig. 3D,E).

#### Baicalein decreased the production of NO and the protein expression of iNOS

**Figure 4:**
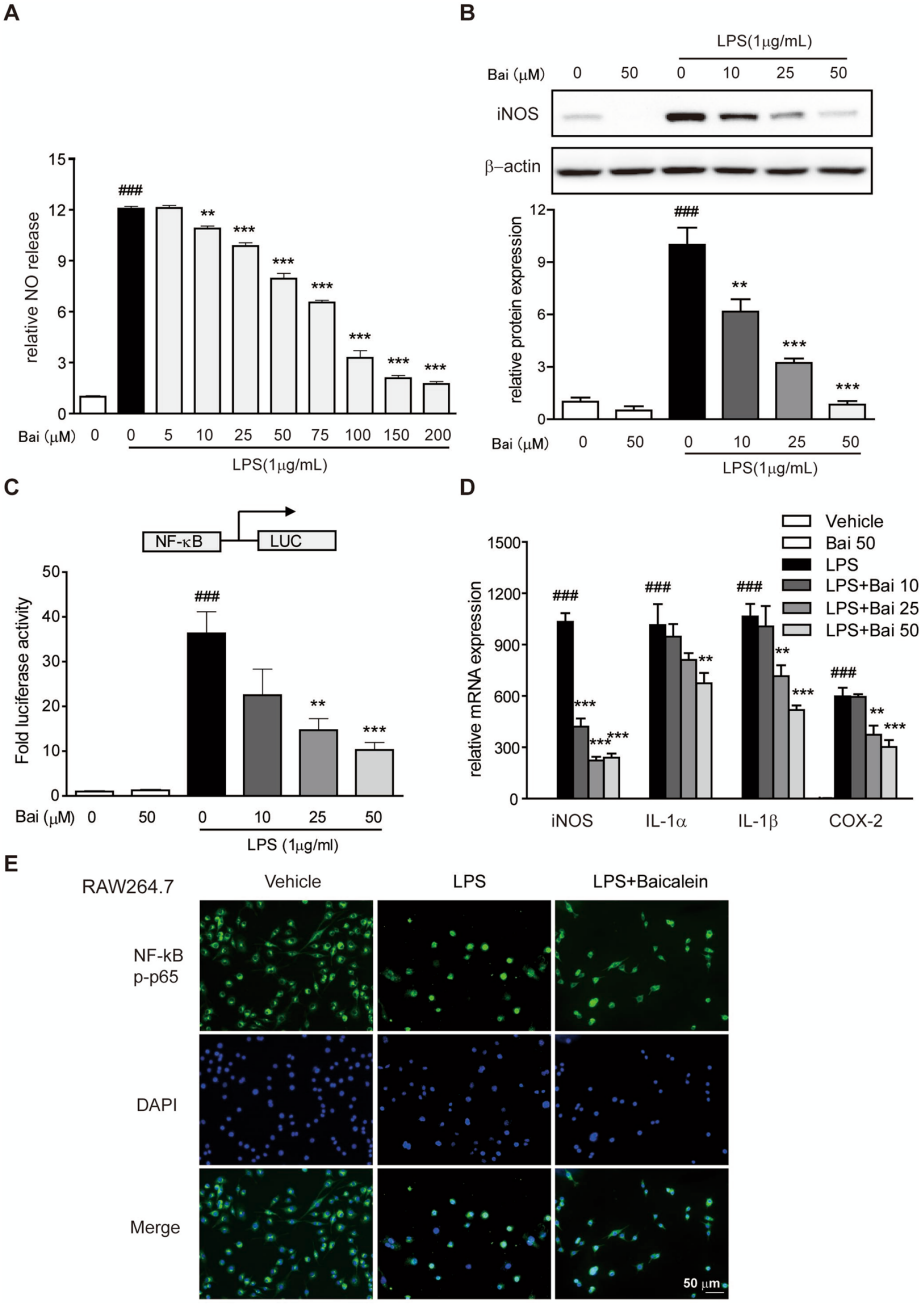
Baicalein inhibited NF-κB pathway *in vitro*. Cells were treated with dose range of baicalein for 2 h prior to LPS (1 µg/ml) treatment for an additional 24 h. (**A**) The production of NO in RAW264.7 cells induced by LPS was measured as described in the Methods. (**B**) Protein level of RAW264.7 cells was determined with antibody against iNOS (1:1000) and β-actin (1:2000 dilution) by immunoblotting. Quantification of the protein expression was performed by densitometric analysis of the blots. Expression was normalized to β-actin. (**C**) NF-κB promoter-driven luciferase activity in RAW264.7 cells was determined using a luciferase assay system as described in the Methods. Results were expressed as fold values of control cells. (**D**) mRNA expression of iNOS, COX-2, IL-1α and IL-1β in THP-1 cells was determined by qRT-PCR. Expression was normalized to β-actin. (**E**) NF-κB p65 nuclear translocation in RAW264.7 cells was evaluated by immunofluorescence staining and images were captured by a fluorescence microscope. Scale bar corresponds to 50 μm and applies throughout. Data were expressed as mean ± SD of three independent experiments (n = 3). ^###^p < 0.001 vs. vehicle-treated group; **p < 0.01, ***P < 0.001 vs. LPS-treated group.

The iNOS gene is known to be the primary regulator of NO production in macrophages^17^. A significant increase in the production of NO and the protein expression of iNOS was observed in RAW264.7 cells exposed to LPS, and treatment with baicalein reduced the production of NO and the protein level of iNOS in a dose-dependent manner (Fig. 4A,B).

#### Baicalein blocked the activation of NF-κB and the pro-inflammatory genes expression

In accord with the *in vivo* data, baicalein inhibited NF-κB-driven luciferase activity induced by LPS in RAW264.7 cells in a concentration-dependent manner (Fig. 4C). On the other hand, the mRNA expression of NF-κB target genes in the LPS-treated THP-1 human macrophage cells, iNOS, COX-2, IL-1α and IL-1β, was dose-dependently inhibited by baicalein (Fig. 4D). Furthermore, the nuclear translocation of NF-κB p-p65 in LPS-stimulated RAW264.7 cells was blocked by baicalein (Fig. 4E).

#### Baicalein suppressed the activation of MAPK signaling molecules

**Figure 5:**
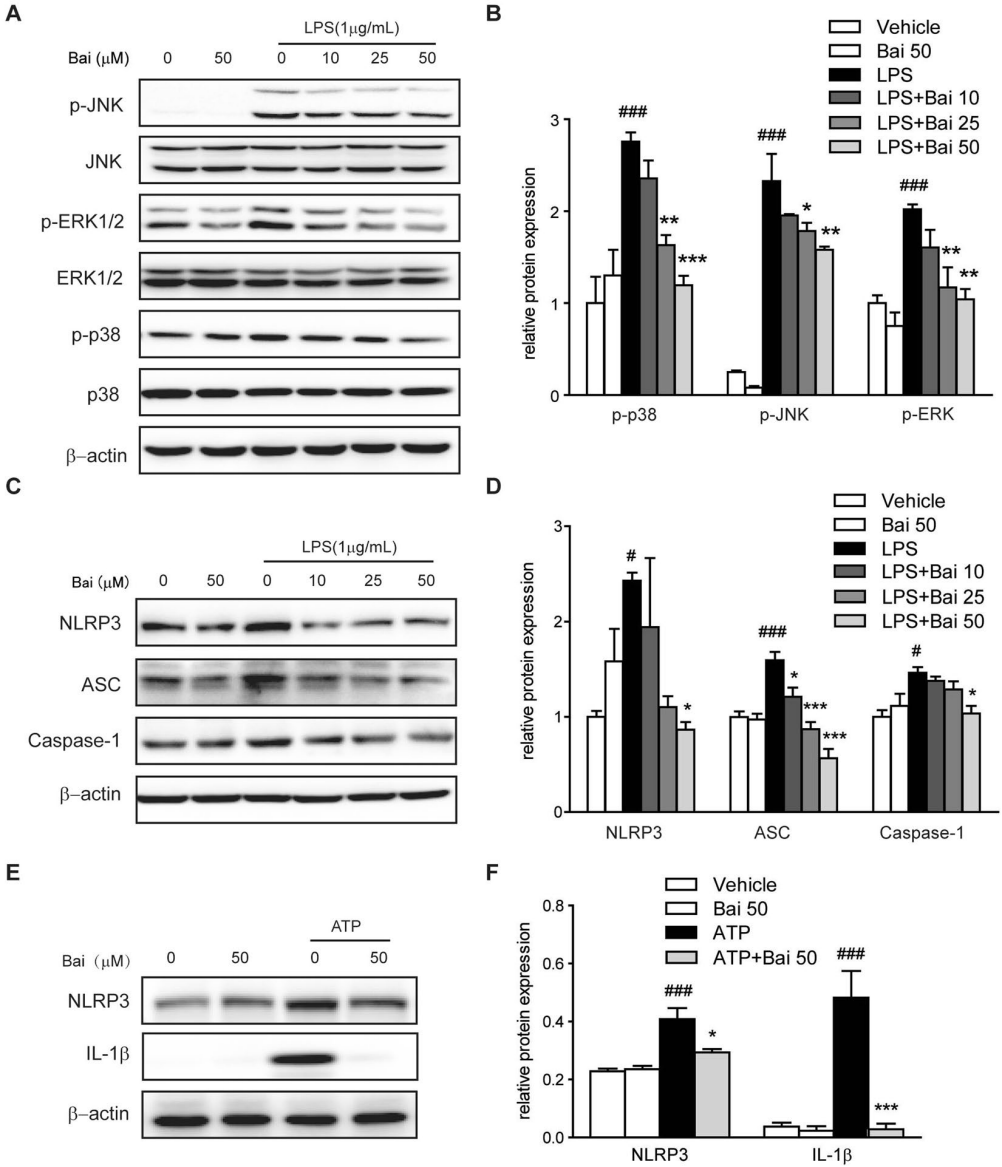
Baicalein inhibited MAPK signaling molecules in RAW264.7 cells and inhibited NLRP3 inflammasome activation in THP-1 cells. Cells were treated with baicalein for 2 h followed by an additional treatment with or without LPS (1 μg/ml) for 24 h. (**A**) Protein levels in RAW264.7 cells were determined with antibodies against JNK, p-JNK, ERK1/2, p-ERK1/2, p38, p-p38 (1:1000 dilution) and β-actin (1:2000 dilution) by immunoblotting. Quantification of the protein expression was performed by densitometric analysis of the blots. The ratio of phosphorylated MAPK to regular MAPK was shown (**B**). (**C**) Protein levels in THP-1 cells were determined with antibodies against NLRP3, ASC, caspase-1 (1:1000 dilution) and β-actin (1:2000 dilution) by immunoblotting. Quantification of the protein expression was performed by densitometric analysis of the blots (**D**). (**E**) THP-1 cells were pretreated with baicalein for 2 h and then followed by stimulation with ATP (5 mM) for 24 h. Protein expression was determined with antibodies against NLRP3, IL-1β (1:1000 dilution) and β-actin (1:2000 dilution) by immunoblotting. Quantification of the protein expression was performed by densitometric analysis of the blots (**F**). Expression was normalized to β-actin. Results were expressed as means ± SD of three independent experiments (n = 3). ^#^p < 0.05, ^###^p < 0.001 vs. vehicle-treated group; *p < 0.05, **p < 0.01, ***P < 0.001 vs. LPS/ATP-treated group.

The activation of MAPK signaling molecules was evaluated by immunoblotting. As shown in Fig. 5A and B, LPS significantly enhanced the phosphorylation of ERK1/2, JNK and p-38 MAPKs in RAW264.7 cells; however, the phosphorylation levels were inhibited by baicalein treatment in a concentration-dependent manner.

#### Baicalein reduced the activation of the NLRP3 inflammasome

Previous studies have suggested that activation of TLR4/NF-κB and TLR4/MAPK signaling pathways contributes to increased IL-1β production^22,23^. Accordingly, the increased mRNA level of IL-1β was observed in the colonic tissues of TNBS-colitis mice (Fig. 2B) as well as in LPS-treated THP-1 human macrophage cells (Fig. 4D). Moreover, the maturation of IL-1β results from the NLRP3 inflammasome activation, which was characterized in our study by increased protein levels of NLRP3, ASC and Caspase-1 in LPS-treated THP-1cells (Fig. 5C,D). The elevated levels of NLRP3, ASC, Caspase-1 and IL-1β induced by LPS were reversed by baicalein treatment in a dose-dependent manner. To investigate whether the decline in the production of IL-1β after baicalein treatment is due to an inhibition in the activation of NLRP3 inflammasome, we examined the protein expression of NLRP3 and IL-1β in THP-1 cells in response to ATP, the NLRP3 inflammasome agonist. The results showed that the relative increase in the protein levels of NLRP3 and IL-1β induced by ATP was decreased by baicalein, suggesting a direct inhibition of NLRP3 inflammasome activation by baicalein (Fig. 5E and F).

#### Docking baicalein to MD-2 pocket revealed the binding affinity

**Figure 6:**
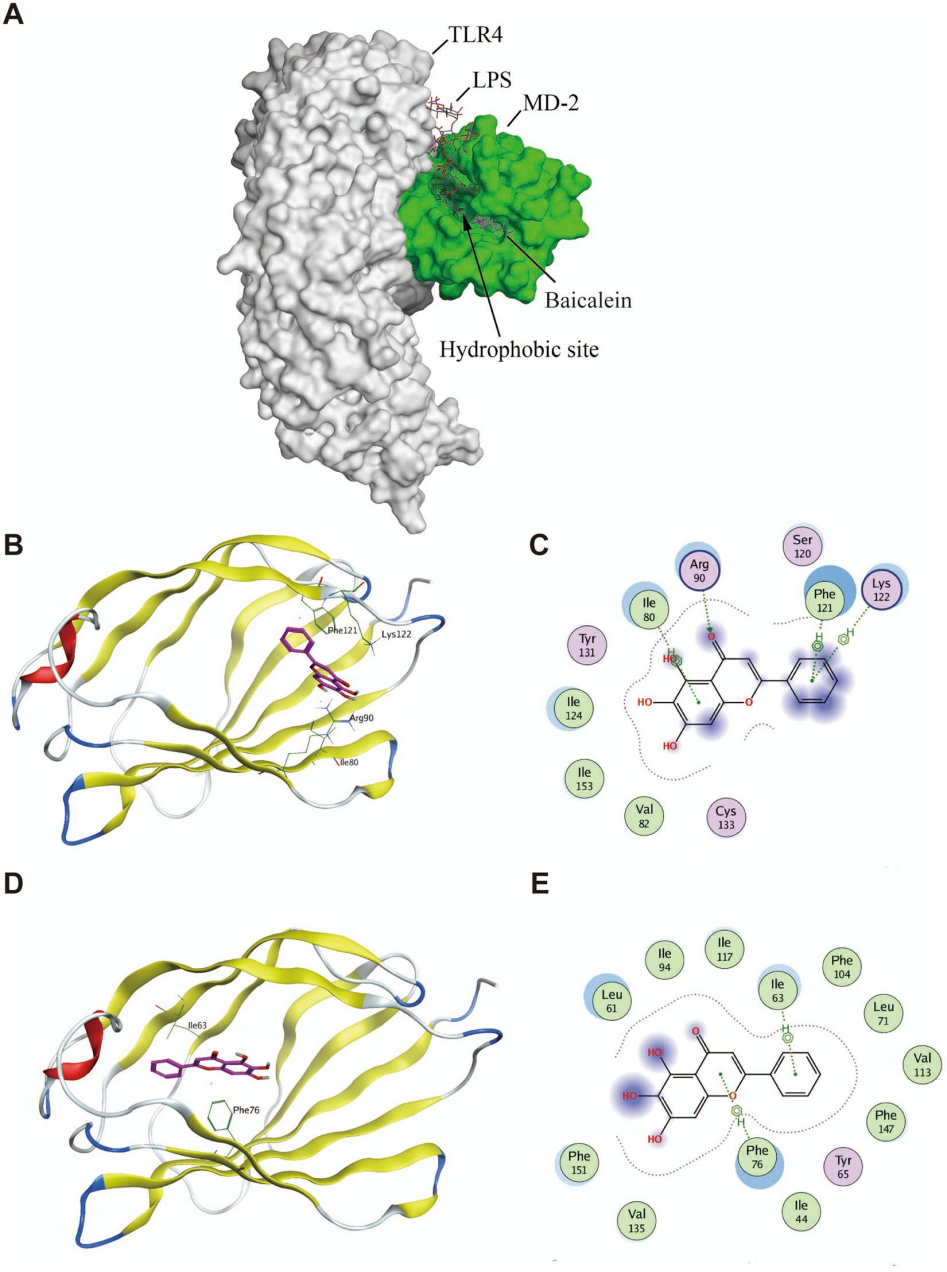
Molecular docking analysis of baicalein to the binding site of MD-2. Baicalein was docked into the hydrophobic pocket of human MD-2 using MOE as described in the Methods. (**A**) The three-dimensional binding pose of baicalein (shown in purple sticks) and LPS (shown in dark grey sticks) in the binding pocket of human MD-2 (shown in green sphere). Among 50 output docking poses, 2 binding conformations were indicated to have the highest docking score: docking mode (**B**) of baicalein (shown in purple sticks) and 2D-interaction schematic diagram (**C**) in the first binding site of MD-2 (shown in ribbon), in which baicalein formed one hydrogen bond with Arg90 and three arene-H interactions with Ile80, Phe121 and Lys122; docking mode (**D**) of baicalein (shown in purple sticks) and 2D-interaction schematic diagram (**E**) in the second binding site of MD-2 (shown in ribbon), in which baicalein formed two arene-H interactions with Ile63 and Phe76.

The TLR4 accessory protein MD-2 is an essential component for recognition of LPS by TLR4^24,25^. LPS binding to the hydrophobic pocket within MD-2 leads to the dimerization of TLR4 and subsequent inflammatory cytokines response^24,25^. To elucidate the underlying mechanisms of baicalein on TLR4 inhibition, we performed molecular docking analysis of baicalein to the binding site of MD-2, in which, baicalein was fitted into the binding pocket of MD-2, displaying close interaction with hydrophobic residues of MD-2. The binding poses of baicalein and LPS in the binding site of human MD-2 was illustrated in Fig. 6A. Among the 50 output docking poses, 2 binding conformations were indicated to have the highest docking score. In the first binding mode, baicalein formed one hydrogen bond with Arg90 and three arene-H interactions with Ile80, Phe121 and Lys122 (Fig. 6B,C). In the second binding mode, two arene-H interactions were formed with Ile63 and Phe76 (Fig. 6D,E). Baicalein was buried inside the hydrophobic pocket in both two binding conformations, indicating overlapping and competition with LPS. These results indicated a possible binding mechanism for baicalein with MD-2 pocket.

#### Baicalein blocked LPS-induced MD-2/TLR4 association

**Figure 7:**
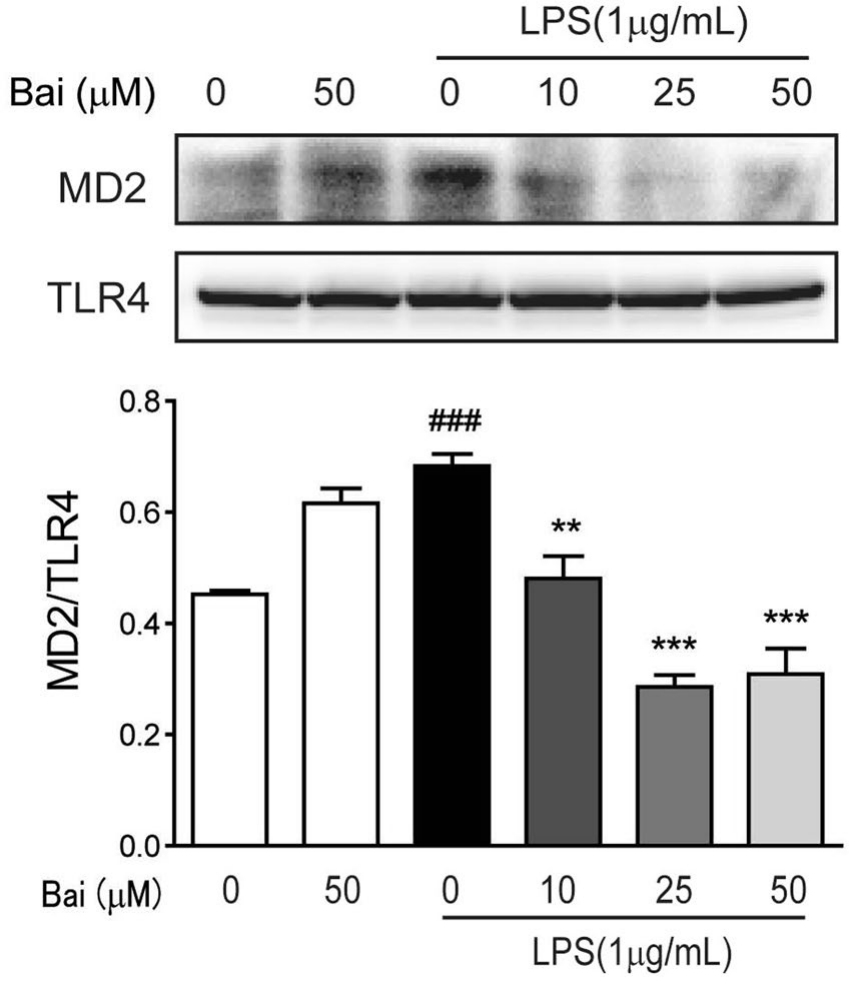
Baicalein blocks LPS-induced MD-2/TLR4 association. RAW264.7 cells were pretreated with baicalein (0, 10, 25, 50 μM) for 2 h and then stimulated with LPS (1 μg/ml) for 24 h. Cell lysates were immunoprecipitated with anti-TLR4 antibody and then subjected to immunoblotting analysis of MD-2 and TLR4. Densitometric analysis of the MD-2/TLR4 ratio in the immunoblots of the immunoprecipitates was carried out. ^###^p < 0.001 vs. the mock group; **p < 0.01, ***P < 0.001 vs. the LPS group.

To evaluate the effect of baicalein on the formation of LPS-induced TLR4/MD-2 complex, co-immunoprecipitation experiment was performed. Briefly, cell lysate was treated with an anti-TLR4 antibody, followed by immunoprecipitation and immunoblot detection of MD-2 and TLR4. As shown in Fig. 7, LPS treatment profoundly increased the co-precipitation of TLR4/MD-2 complex, while treatment with baicalein significantly reduced the LPS-induced TLR4/MD-2 complex in a concentration-dependent manner. These data indicated that baicalein blocked the formation of LPS-induced TLR4/MD-2 complex through binding to MD-2 pocket.

## Discussion

TNBS-induced colitis is a well-established experimental model with features resembling human CD, such as progressive weight loss, bloody diarrhea, large bowel wall thickening, diffuse necrosis, intense transmural inflammation and neutrophils infiltration^14,15^. In the current study, we have demonstrated that baicalein could ameliorate the disease symptoms of TNBS-induced colitis, including body weight loss, diarrhea, colon shortening and histological injury. Notably, none of the mice receiving baicalein alone exhibited apparent body weight loss, diarrhea, colon shortening and mucosal disruption throughout the study, which indicates the relative safety of baicalein management. In addition, the TNBS-induced MPO activity, an indicator of neutrophil influx and transmural inflammation^18^, was decreased in baicalein-treated mice. On the other hand, extensive studies have demonstrated that the increased pro-inflammatory cytokines and chemokines play important roles in the pathogenesis of CD^21^. According to our data, baicalein effectively inhibited the mRNA expression of iNOS, ICAM-1, COX-2, MCP-1, TNF-α and IL-1β in the colon of TNBS-colitis mice.

Although the underlying pathogenesis of IBD remains unclear, dysregulation of immune reaction to intestinal bacterial flora has been implicated in the development of IBD. The innate immune system recognizes the presence of specific bacterial antigens through an extensive family of pattern recognition receptors (PRRs)^26^. TLR4 is a PRR that recognizes LPS, a major component of the outer membrane of gram-negative bacteria, and activates the secretion of pro-inflammatory mediators, leading to the inflammatory response. Additionally, TLR4 expression is increased in colonic mucosa from mice with experimental colitis as well as in intestinal epithelial cells from patients with IBD^27,28^. Compelling evidence indicates the beneficial effect of suppressing TLR4/MyD88 signaling molecules, finally leading to the inactivation of NF-κB and MAPKs, and the inhibition of pro-inflammatory cytokines^23,29^. In this study, we showed that baicalein significantly inhibited the up-regulation of TLR4 and MyD88 in TNBS-induced colitis mice and in LPS-induced macrophages. The TNBS or LPS-induced activation of NF-κB and MAPKs was inhibited by baicalein treatment. Meanwhile, baicalein decreased the expression of COX-2, IL-1α and IL-1β induced by LPS in a concentration-dependent manner. The stimulation of LPS activated the secretion of NO and the expression of iNOS, while all these alterations were inhibited by baicalein in a concentration-dependent manner. These findings indicated that down-regulation of TLR4/MyD88 signaling cascades (NF-κB and MAPK) was involved in the anti-inflammatory effects of baicalein in TNBS-induced colitis and in LPS-induced macrophages.

However, activation of TLR4 by LPS requires an accessory protein MD-2, since most of the lipid chains of LPS interact with the hydrophobic pocket of MD-2^24,25^. Recognition of LPS by MD-2 triggers the formation of TLR4/MD-2 complex, which leads to the recruitment of one major adaptor, MyD88, and the activation of NF-κB and MAPK pathways^24^. Several studies have shown that some natural and synthetic chemicals bind directly to the MD-2 pocket and block the recognization of TLR4 by LPS, leading to inflammatory signaling interception^30,31^. To identify the underlying molecular target of baicalein in TLR4 signaling, we tested whether baicalein could bind to MD-2 and interfere with the interaction between LPS and TLR4/MD-2 receptor complex. According to the results of molecular docking, baicalein embedded into the hydrophobic pocket of MD-2 and overlapped with the LPS-binding site in MD-2. We further examined the effects of baicalein on LPS-induced MD-2/TLR4 association by co-immunoprecipitation assay. The results showed that baicalein inhibited the association between MD-2 and TLR4 during LPS treatment in a concentration-dependent manner. To our knowledge, for the first time, we demonstrated that baicalein blocked the formation of the LPS-induced TLR4/MD-2 complex through binding to MD-2 pocket. These findings indicated that baicalein could directly bind to MD-2 to block MD-2/TLR4 association, resulting in the inhibition of the downstream signaling cascades (NF-κB and MAPKs).

The production of pro-inflammatory cytokines is governed not only by TLR4/MyD88 signaling pathway but also by a large multimeric protein complex known as inflammasome^22,23^. The inflammasome complex is typically composed of three components, namely: NLR, ASC and caspase-1. Several inflammasomes have been described, of which the NLR family, NLRP3 inflammasome is the most investigated^23^. Upon activation, NLRP3 recruits ASC adaptor, which in turn promotes the recruitment of pro-caspase-1. Pro-caspase-1 then clusters and autocleaves to generate enzymatically active caspase-1. Activation of caspase-1 is required to convert pro-IL-1β to its mature active form IL-1β. It is known that IL-1β is mainly produced by LPS-activated macrophages via the activation of the NLRP3 inflammasome, and it is increased in mucosal tissue of CD patients^32,33^. Given the critical role of NLRP3 inflammasome in intestinal homeostasis and colitis, THP-1 human macrophage cell line was used as a model to investigate the effects of baicalein on NLRP3 inflammasome^22^. We observed that IL-1β was increased in both TNBS-induced colitis mice and LPS-induced THP-1 human macrophage cells. Baicalein effectively reduced the level of IL-1β, which was accompanied by the inhibition of NLRP3, ASC and caspase-1 in a dose-dependent manner.

Several previous studies have shown that baicalein down-regulates pro-inflammatory molecules. Ku *et al*. reported that baicalein prevents from high glucose-induced vascular inflammation via suppressing the formation of reactive oxygen species (ROS), the expression of cell adhesion molecules (CAMs), and the activation of NF-κB^34^. Moreover, pre-administration of baicalein in mice is indicated to prevent from cisplatin-induced acute renal injury via up-regulation of antioxidant defense and down-regulation of the MAPK and NF-κB signaling pathways^35^. Furthermore, baicalein has been reported to have anti-tumor activity in various cancer models^36^. Treatment with baicalein has been shown to inhibit cell proliferation, induce apoptosis, activate PPARγ, and inhibit NF-κB pathway in inflammation-associated colonic cancer models^7^. Therefore, the anti-tumor properties of baicalein increase its promising medicinal value for patients with long-standing IBD, because the most severe clinical complication for patients with long-standing IBD is the development of colonic cancer^21^.

Collectively, these novel findings demonstrated that baicalein ameliorated TNBS-induced colitis. TLR4/MyD88 pathway and its downstream signaling molecules, NF-κB and MAPKs, played major roles in TNBS-induced production of pro-inflammatory cytokines and mediators, while the NLRP3 inflammasome was required for the secretion of IL-1β. Since baicalein is a natural compound with little toxicity in our experimental models, our findings may contribute to the effective utilization of baicalein or its derivatives in the treatment of human IBD.
